# Rusty pipe syndrome: a case report and review of the literature

**DOI:** 10.1186/s12884-022-05048-5

**Published:** 2022-10-13

**Authors:** Huanna Tang, Wenting Zhu, Jianpeng Chen, Dan Zhang

**Affiliations:** 1 Key Laboratory of Reproductive Genetics (Ministry of Education) and Department of Reproductive Endocrinology, Women’s Hospital, Zhejiang University School of Medicine, Hangzhou, 310006 Zhejiang China; 2 Key Laboratory of Women’s Reproductive Health Research of Zhejiang Province and Department of Reproductive Endocrinology, Women’s Hospital, Zhejiang University School of Medicine, Hangzhou, 310006 Zhejiang China

**Keywords:** Bloody discharge, Rusty pipe syndrome, Breastfeeding, Case report

## Abstract

**Background:**

Painless bloody nipple discharge is often classified as pathological due to its association with malignant lesions. However, it can also be a completely harmless condition. Rusty pipe syndrome is a rare cause of benign, self-limiting bloody nipple discharge during late pregnancy and early lactation. Given that rusty pipe syndrome is not described in conventional textbooks, we thought it would be appropriate to bring this benign disease to the notice of readers.

**Case presentation:**

A 31-year-old G1P1 female delivered an infant with a birth weight of 3000 g via cesarean section at 39 weeks of gestation. The baby was admitted to the pediatric intensive care unit for a suspected oblique inguinal hernia. The mother had bilateral painless bloody nipple discharge when she started to express milk. A physical examination uncovered no signs of inflammation, engorgement, palpable mass, tenderness, cracks or ulcers. A breast ultrasound and cytological analysis revealed no signs of a neoplasm. Without any medical intervention, the color of the rusty milk changed from dark brown to light brown during hospitalization and finally resolved six days postpartum.

**Conclusion:**

Rusty pipe syndrome is a self-limiting benign condition that should be considered in the differential diagnosis of bloody nipple discharge. Awareness of this rare disease by medical professionals would be extremely beneficial for avoiding unneeded examinations and discontinuity of exclusive breastfeeding.

## Background

Human milk is considered the gold standard for infant feeding [1]. However, several problems can occur during the lactation period and compromise the breastfeeding rate. In clinical practice, a bloody nipple discharge during pregnancy or lactation is a rarely encountered symptom that results in significant patient worry and prompts medical evaluation and intervention [2]. Rusty pipe syndrome (RPS) is a benign physiological condition that occurs in primiparous women. RPS typically presents with brown or bloody milk mimicking flowing water from a rusty pipe; it is usually bilateral, painless, and self-limiting [3]. Most cases will clear within seven days without any medical treatment [4–16]. There are not many case reports on this topic, and its occurrence in China is quite rare. Due to the underrecognized and underdiagnosed condition of RPS, we conducted a review of blood-stained discharge of the breast and reported a case of RPS in a 31-year-old woman to achieve several objectives: first, to identify the clinical manifestation and lab characteristics of RPS; second, to discuss what doctors should do when faced with self-healable bloody nipple discharge when there are no masses in the breasts and what evidence is required before making a diagnosis.

## Case report

**Fig. 1:**
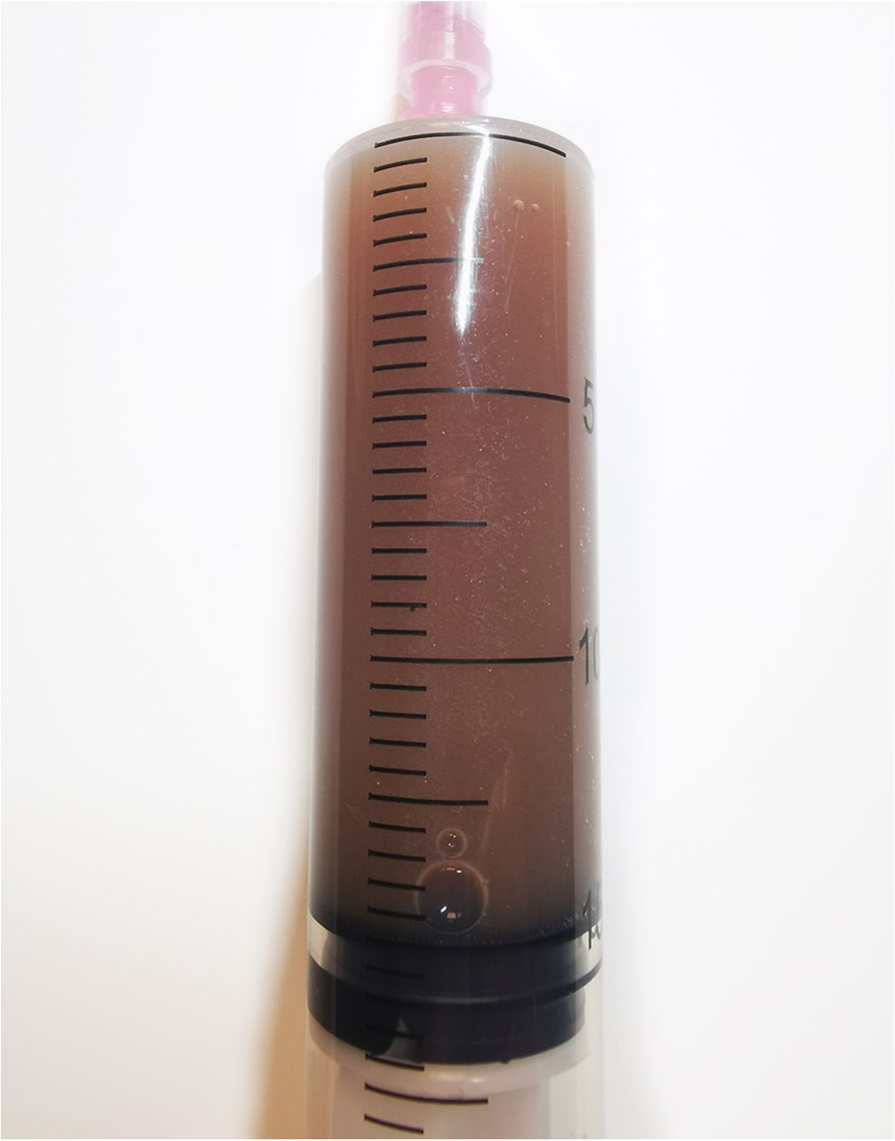
Breast milk color of the patient with Rusty pipe syndrome (camera, HUAWEI P30pro)

**Fig. 2:**
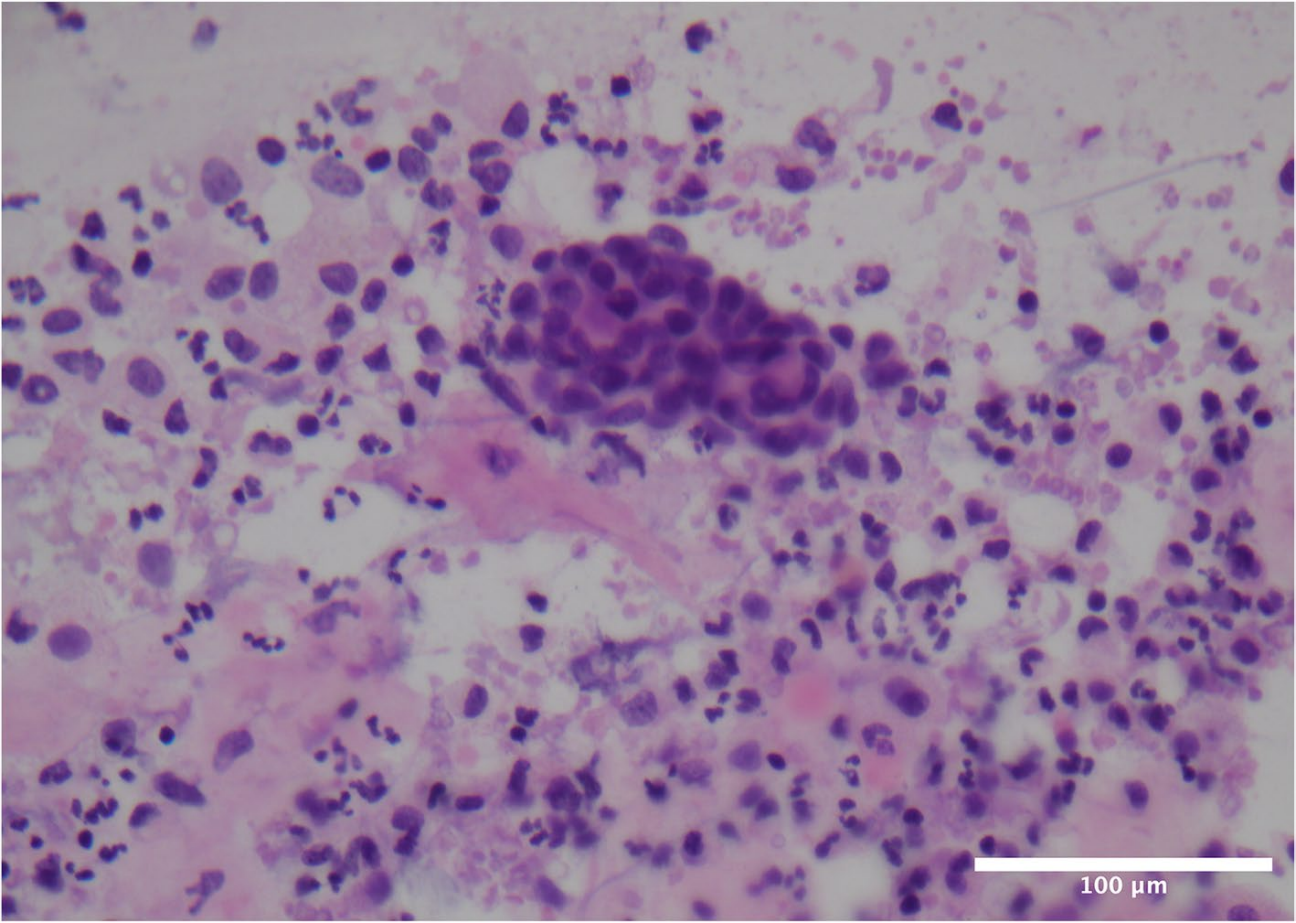
Cytological smear of nipple discharge showing mammary epithelial cells and inflammatory cells (hematoxylin–eosin stain; microscope, LEICA DM2500/ × 40; software, NIS-Elements F 4.60.00 64-bit; camera, Nikon DS-Fi3;scale bars, 100 μm)

A 31-year-old primipara delivered an infant with a birth weight of 3000 g via cesarean section at 39 weeks of gestation. After delivery, the infant was transferred to the neonatal intensive care unit after being diagnosed with an oblique inguinal hernia. Due to the separation of the mother and baby, the patient could only express milk and send it to the baby. The mother noticed a bilateral painless bloody nipple discharge when she expressed milk (Fig. 1). She told the doctor that this phenomenon occurred as early as 36 weeks of pregnancy. She denied experiencing any infection or trauma, and reported no suckling by the baby. The patient had no family history of any breast disease. She was in good health and denied having any alcohol or drug during pregnancy. A physical examination found no signs of inflammation, engorgement, palpable mass, or tenderness on either breast, and no cracks or ulcers on the nipples. Breast ultrasound imaging revealed lobular hyperplasia and mild ductal ectasia with no breast cyst, solid nodule, or enlarged lymph nodes. Cytological analysis of the milk showed large numbers of inflammatory cells, polynuclear macrophages, and a small number of epithelial cells with no signs of atypia (Fig. 2). A surgical consultation was obtained, and intraductal papilloma was ruled out as a cause because the blood-stained breastmilk was bilateral and emanated from multiple ducts. The patient was advised to express milk from her breasts every three hours to avoid breast engorgement and support continued breastfeeding. The rusty milk color changed from dark brown to light brown during hospitalization and finally resolved spontaneously six days after delivery, with no recurrence. Additional formula milk was given to the baby at first, but at discharge on the sixth day, the baby was exclusively breastfed. The mother-infant pair was followed up in the postpartum clinic for six months. Postpartum follow-up has revealed no evidence of neoplastic changes thus far. This case report follows the CARE Guidelines. Informed consent was obtained from the patient for publication.

## Discussion and conclusions

Bloody nipple discharge tends to coincide with symptomatic dysfunction of the ductal system and the potential presence of a subclinical malignancy in nonpregnant women [17]. Many authors have studied the relationship between bloody nipple discharge and breast carcinoma, observing that malignancy occurs in 8–30% of women with bloody discharge [18–24]. The occurrence of blood-stained nipple discharge is rare during pregnancy, and its prevalence is estimated to be approximately 0.1% [25]. It may cause great anxiety to the patient because of the perceived link with breast carcinoma in nonpregnant patients [26]. However, blood-stained milk may occur as a result of physiological changes during late pregnancy and the early lactation period. One such condition can be RPS, a self-limiting condition that presents as painless bloody or brown discharge in pregnant and postpartum women. Bloody nipple discharge correlates with proliferation of the duct epithelium. The capillary network around mammary ducts is fragile due to hormonal stimuli (estrogen, progesterone, oxytocin) and is easily traumatized, resulting in blood cells leaking into breast secretions [12, 16, 27].

To date, only 16 cases diagnosed with RPS have been reported (including one in this paper) [3–16]. A review of RPS cases including clinical manifestations and auxiliary examinations are summarized in Table 1. As shown in Table 1, the abnormal discharge was bilateral in all patients. Among those cases, 15 patients were primiparas, while one patient was a secundipara. For the secundipara described by Faridi [13], brown–red colored milk occurred both in her first and second pregnancies, while the other cases only had one occurrence. In the literature, RPS usually presents in the second or third trimester, or within the first few days of lactation: four mothers observed a bloody discharge several weeks before delivery (from the 26^th^ to 36^th^ week of gestation), while another 11 mothers started to have blood-stained milk within two days postnatally. The abnormal color of the milk faded away spontaneously in a progressive manner and completely disappeared in ten days. A follow-up of these patients ranging from six weeks to six months revealed no evidence of neoplastic changes on physical examination. Lactation was achieved successfully in all women who intended to breastfeed.

**Table 1:** Summary of cases in the literature

Literature	Age	G/P	GW (weeks)	Bilateral or unilateral	Time of onset	Resolution of bleeding	Cytology	Imaging results
This paper	31	1/1	39	bilateral	36th week at pregnancy	6 day postnatal	inflammatory cells, polynuclear macrophages and epithelial cells	lobular hyperplasia and mild ductal ectasia
Katarzyna et al.2022 [3]	29	1/1	40	bilateral	day 1 after delivery	5 day postnatal	amorphous acidophilous content, mononuclear macrophages, erythrocytes	NA
Low et al.2021 [4]	30	1/1	NA	bilateral	first hour after delivery	5 day postnatal	NA	NA
Kural et al.2020 [5]	28	1/1	40	bilateral	day 2 after delivery	within 72 h	NA	NA
Anil et al.2020 [6]	23	1/1	NA	bilateral	first hour after delivery	4 day postnatal	NA	normal
Mohamad et al.2020 [7]	29	1/1	38	bilateral	NA	6 day postnatal	normal	bilateral simple breast cyst
Francini et al.2018 [8]	29	1/1	38	bilateral	first hour after delivery	within 24 h	NA	sparse bilateral breast cyst
Ersin et al.2017 [9]	28	1/1	33	bilateral	first hour after delivery	7 day postnatal	normal	normal
Barco et al.2014 [16]	26	1/1	40	bilateral	32nd week at pregnancy	10 day postnatal	Red blood cells, histiocytes and foam cells	dense breast tissue, mild duct ectasia
Silva et al.2014 [10]	31	1/1	39	bilateral	first hour after delivery	4 day postnatal	normal	normal
Cizmeci et al.2013 [11]	28	1/1	40	bilateral	day 2 after delivery	within 72 h	NA	NA
Usharani et al. 2013 [12]	21	1/1	NA	bilateral	12 h after delivery	5 day postnatal	NA	NA
Faridi et al.2013 case1 [13]	28	2/2	38	bilateral	first hour after delivery	7 day postnatal	red blood cells, foamy macrophages	normal
Faridi et al.2013 case2 [13]	27	1/1	37	bilateral	7 month at gestation	7 day postnatal	red blood cells	NA
Moreau et al.2013 [14]	22	1/1	40	bilateral	26 week at gestation	4 day postnatal	normal	dilated ducts
Virdi et al.2001 [15]	26	1/1	NA	bilateral	20 h after delivery	within 48 h	normal	NA

*Abbreviations: NA* Not available, *G/P* Gravidity/parity, *GW* Gestational weeks

RPS can mimic more serious diseases, such as breast cancer. To minimize the rate of misdiagnosis, it is necessary to carefully assess and thoroughly understand the appearance of various benign and malignant breast lesions. A thorough physical examination may quickly differentiate conditions such as trauma, broken nipple, and mastitis. The most common cause of pathological nipple discharge is ductal papilloma, which accounts for 35–57% of cases, followed by duct ectasia (17–36%) and malignant lesions (4–21%) [28, 29]. Pregnancy-associated breast cancer (PABC) is defined as breast cancer diagnosed during pregnancy or in the first year of lactation. Most patients with PABC present with an asymptomatic mammary mass (not everyone does), and bloody nipple discharge, breast swelling, and pain are less frequent symptoms. King et al. reported on 63 cases of PABC, and three of them exhibited bloody nipple discharge [30]. Bloody nipple discharge and/or nipple retraction were reported in 8% of PABC patients in a study by Taskin et al. [31]. Wang et al. evaluated 142 patients with PABC, four of whom presented with papillary hemorrhagic discharge [32]. However, little information was available in the cases described by King, Taskin, and Wang. Disease status, time of onset, and discharge location were all lacking. Intraductal papilloma is often associated with unilateral, painless, bloody or serous nipple discharge in premenopausal women and is rarely observed in pregnant women. Cheah et al. described a patient presenting with a left breast mass (later identified as a benign papilloma) with bloody nipple discharge at 20 weeks of gestation [33]. Intraductal papilloma should be considered a differential diagnosis and excluded to diagnose RPS. Patients with mammary duct ectasia typically present with intermittent nipple discharge, swelling, and mild erythema near the areola [34]. Notwithstanding the extremely rare coexistence of pregnancy and the abovementioned diseases, such pathological conditions should be ruled out before RPS is diagnosed.

The first diagnosis of RPS is established by the history of the present illness and regular physical examination, followed by specific tests, such as cytological analysis of the bloody discharge and breast ultrasonography, if necessary. The available literature offers a mixed opinion on whether obtaining a cytological smear is strictly required. The diagnostic accuracy of nipple discharge fluid cytology for the detection of breast cancer is poor, while specificity and sensitivity data vary among published clinical studies. A meta-analysis from Jiwa et al. indicated that the sensitivity of nipple smear cytology was approximately 75% with a specificity of 87% [35]. Nipple discharge cytology is deemed difficult to diagnose given the presence of atypical cellular changes unrelated to a malignancy and the variability of interpretation by pathologists. Even though the clinical utility of cytology is limited, researchers discovered that the diagnostic accuracy of nipple smear cytology is similar to that of other available diagnostic methods, such as breast ultrasound and MRI [35]. Regarding imaging, the most suitable radiologic approach for assessing breast problems in pregnant and lactating women is ultrasound [27, 36]. The physiological changes of lactating breasts present as lobular hyperplasia and ductal ectasia, resulting in the sonographic appearance of large, hypoechoic ducts and lobules on a background of diffusely reduced breast echogenicity [37, 38]. Ultrasound images obtained from patients with RPS show similar physiological characteristics [6–8, 14, 16]. All masses discovered during pregnancy and breastfeeding should be thoroughly assessed, since nonrelevant or physiological masses caused by hormonal stimulation can only be diagnosed after a thorough radiologic examination [39]. Thus, patients presenting with unremitting bloody nipple discharge should urgently undergo a triple assessment (clinical, imaging, and pathological findings) due to the increased prevalence of breast cancer. Unless the lump is growing quickly or there is discordance in the triple assessment, a core needle biopsy or surgery is not recommended in pregnant or lactating women.

A variety of management approaches are reported in our review, ranging from reassurance only to temporarily discarding expressed breast milk and using formula instead. If the infant tolerates the milk, breast feeding is encouraged during this period. The prognosis of RPS is good; the blood-stained discharge should clear within seven days for most cases, and only one report showed bloody milk fully resolved by day ten [16]. The patients should be followed up regularly at least until the milk color is normal. If bloody milk continues for more than one week, further investigation, such as ultrasound and cytology analysis, should be carried out to rule out pathological causes. Long-term clinical follow-up appears normal on physical examination with no evidence of neoplastic alterations [5, 13, 14, 16]. The presence of RPS would not have a poor prognosis for neonates either. The results of the current follow-up show that breastfed babies are free from diseases such as growth retardation and breast milk intolerance [3, 5, 13]. Early diagnosis of this rare, self-limiting disease by obstetricians or neonatologists, followed by telling the mother that her infant would be unaffected by the small quantity of blood consumed, would be extremely beneficial in avoiding unneeded examinations and the discontinuation of exclusive breastfeeding.

## Data Availability

All data generated for this study are available on request to the corresponding author.

## Abbreviations

RPS: Rusty pipe syndrome
G1P1: Gravida1 para1
PABC: Pregnancy-associated breast cancer
